# A complement component C1q-mediated mechanism of antibody-dependent enhancement of Ebola virus infection

**DOI:** 10.1371/journal.pntd.0008602

**Published:** 2020-09-04

**Authors:** Wakako Furuyama, Asuka Nanbo, Junki Maruyama, Andrea Marzi, Ayato Takada

**Affiliations:** 1 Laboratory of Virology, Division of Intramural Research, National Institute of Allergy and Infectious Diseases, National Institutes of Health, Rocky Mountain Laboratories, Hamilton, Montana, United States of America; 2 Department of Cell Physiology, Hokkaido University Graduate School of Medicine, Sapporo, Japan; 3 Division of Global Epidemiology, Research Center for Zoonosis Control, Hokkaido University, Sapporo, Japan; 4 Global Station for Zoonosis Control, Global Institution for Collaborative Research and Education, Hokkaido University, Sapporo, Japan; 5 School of Veterinary Medicine, the University of Zambia, Lusaka, Zambia; University of Texas Medical Branch / Galveston National Laboratory, UNITED STATES

## Abstract

Besides the common Fc receptor (FcR)-mediated mechanism of antibody-dependent enhancement (ADE), Ebola virus (EBOV) is known to utilize the complement component C1q for ADE of infection. This mechanism is FcR-independent and mediated by cross-linking of virus-antibody-C1q complexes to cell surface C1q receptors, leading to enhanced viral entry into cells. Using confocal microscopy, we found that virus-like particles (VLPs) consisting of EBOV glycoprotein, nucleoprotein, and matrix protein attached to the surface of human kidney 293 cells more efficiently in the presence of an ADE monoclonal antibody and C1q than with the antibody or C1q alone, and that there was no significant difference in the efficiency of VLP uptake into endosomes between the C1q-mediated ADE and non-ADE entry. Accordingly, both ADE and non-ADE infection were similarly decreased by inhibitors of the signaling pathways known to be required for endocytosis. These results suggest that C1q-mediated ADE of EBOV infection is simply caused by increased attachment of virus particles to the cell surface, which is distinct from the mechanism of FcR-mediated ADE requiring intracellular signaling to promote phagocytosis/macropinocytosis.

## Author summary

Ebola virus (EBOV) utilizes the complement component C1q for antibody-dependent enhancement (ADE) of infection. We found that an ADE antibody increased viral attachment in the presence of C1q and that there was no significant difference in the efficiency of viral uptake into endosomes between the C1q-mediated ADE and non-ADE entry. Accordingly, both ADE and non-ADE infection were similarly decreased by inhibitors of the signaling pathways for endocytosis. These results suggest that C1q-mediated ADE of EBOV infection is simply caused by increased viral attachment to the cell surface, most likely via cross-linking of virus-antibody-C1q complexes to cellular C1q receptors.

## Introduction

Ebola virus (EBOV), a member of the family *Filoviridae*, causes severe hemorrhagic fever in humans and nonhuman primates with human case fatality rates of up to 90% [1]. EBOV expresses a single transmembrane glycoprotein (GP) that is responsible for both receptor binding and membrane fusion [2, 3]; thus, the GP is the only known target of neutralizing antibodies against EBOV. Some of the GP-specific antibodies are known to increase the entry of EBOV into cells, causing antibody-dependent enhancement (ADE) of infection *in vitro* [4–7]. Previous studies have shown that convalescent human sera contain ADE antibodies [4, 7], raising concerns about potential detrimental effects for a second EBOV infection or passive immunization with convalescent human serum, which is currently under consideration as a treatment of EBOV disease.

Two distinct pathways of EBOV ADE are known; Fc receptor (FcR)-mediated and complement component C1q-mediated ADE [4, 8]. We previously demonstrated that intracellular signaling pathways promoting phagocytosis and/or macropinocytosis play a key role in FcR-mediated ADE [9]. It is also known that the presentation of C1q induces enhanced phagocytic activity [10] and that C1q binds C1q receptors expressed on many different cell types and triggers signaling pathways such as Wnt/β-catenin, PI3K, and some tyrosine kinases [11–13]. However, potential roles of these signaling pathways in C1q-mediated ADE remain elusive.

To determine the molecular mechanisms underlying C1q-mediated ADE of EBOV infection, we focused on C1q- and FcR-dependent signaling and found that these signaling cascades were not specifically important for the C1q-mediated ADE entry into cells. Our data suggest that the increased viral attachment to the cell surface occurs via crosslinking of antibody, C1q, and C1q receptors leads to C1q-mediated ADE of EBOV infection.

## Methods

### Cells and viruses

Human embryonic kidney 293 (HEK293) and Vero E6 cells were grown in Dulbecco’s modified Eagle’s medium (DMEM) (D5796; Sigma) supplemented with 10% fetal calf serum (FCS) (Cell Culture Bioscience), 100 U/ml penicillin, and 0.1 mg/ml streptomycin (Gibco). These cells were obtained from an already-existing collection in the Research Center for Zoonosis Control, Hokkaido University. Replication-incompetent vesicular stomatitis virus (VSV) pseudotyped with EBOV GP containing GFP instead of the VSV G gene (VSVΔG-EBOV GP) was generated as described previously [3, 14]. Titers of the pseudotyped VSVs were determined as infectious units (IUs) by counting the number of GFP-positive cells. Replication-incompetent pseudotyped VSV with the VSV glycoprotein (VSVΔG-VSV G) was used as a control virus.

### ADE assays

VSVΔG-EBOV GP appropriately diluted to yield 50–100 IUs/50 μl in HEK293 or Vero E6 cells were incubated for 1 h at room temperature with 80, 20, 5, 1.25, or 0.31 μg/ml C1q (C1740; Sigma) and 1 μg/ml EBOV GP-specific mouse monoclonal antibody (MAb) ZGP12/1.1 (IgG2a), which is known to be an ADE antibody that enhances EBOV infection *in vitro* [5], and then inoculated onto confluent monolayers of HEK293 cells. MAb S139/1 (IgG2a) specific to influenza A virus hemagglutinin was used as a negative control (CTR) IgG antibody [15]. Twenty-four hrs later, GFP-positive cells were counted using an IN Cell Analyzer 2000 (GE Healthcare) or Immunospot S6 ULTIMATE Analyzer (Cellular Technology Limited). To reduce the background (i.e., residual) infectivity of the parent VSVΔG-VSV G, the VSVΔG-EBOV GP stock supernatant was treated with a neutralizing MAb to the VSV G protein (VSV-G[N]1–9) before use.

### Inhibitor and antibody treatments

For infection assays, HEK293 cells were treated with Wnt/β-catenin signaling pathway inhibitors BMS-777607 (Selleckchem), IWP-2 (Selleckchem), or LGK-974 (Selleckchem), spleen tyrosine kinase inhibitor R788 (Santa Cruz), sarcoma family protein-tyrosine kinase inhibitor PP2 (Tocris), Mer tyrosine kinase UNC2250 (Selleckchem), Bruton’s tyrosine kinase inhibitor LFM-A13 (Focus Biomolecules), phosphatidylinositol 3-kinase inhibitor LY294002 (Wako), rat sarcoma inhibitor Manumycin A (Santa Cruz), or anti-GC1q receptor (GC1qR) (clone 60.11; Abcam) at the indicated concentrations for 1 h at 37˚C and then infected with VSVΔG-EBOV GP or VSVΔG-VSV G. The relative percentage of infectivity was calculated by setting the IU value of the CTR-IgG+C1q- or ZGP12/1.1+C1q-treated VSVΔG-EBOV GP or non-treated VSVΔG-VSV G in DMSO or ethanol-treated cells to 100%. Cell viability was determined using trypan blue staining (BIO-RAD).

### Western blot analysis

HEK293 or Vero E6 cells resuspended in phosphate-buffered saline (PBS) were mixed with equal volume of sodium dodecyl sulfate-polyacrylamide (SDS) gel electrophoresis sample buffer containing 2.86 M β-mercaptoethanol (Sigma) and heated to 99 °C for 10 min. SDS-PAGE was performed and separated proteins were transferred to a Trans-Blot polyvinylidene difluoride membrane (Bio-Rad Laboratories) as described elsewhere [16]. The membrane was blocked for 3 h at room temperature with 3% skim milk. After washing with phosphate-buffered saline containing 0.1% Tween (PBST), the membrane was incubated with one of the following rabbit or mouse antibodies for overnight at 4 °C: 4 μg/ml anti-GC1qR (clone 60.11; Abcam), anti-CD93 1:1,000 (Abcam), anti-CD35 1:1,000 (Abcam), 2 μg/ml anti-low-density lipoprotein receptor-related protein 1 (LRP-1) (PROGEN) in PBST containing 1% skim milk. After washing 3 times with PBST, the membrane was incubated with horse-radish peroxidase (HRP)-labeled secondary antibodies, anti-mouse IgG (1:10,000) or anti-rabbit IgG (1:5,000) (Jackson ImmunoResearch) in PBST containing 1% skim milk for 1 h at room temperature. After washing 3 times with PBST, the bound antibody was visualized with the SuperSignal West Pico chemiluminescent substrate (Thermo Fisher Scientific). Images were analyzed by an iBright CL1500 Imaging System (Thermo Fisher Scientific).

### Imaging of attachment and trafficking to late endosomes of lipophilic tracer (DiI)-labeled virus-like particles (VLPs)

For imaging of viral attachment and trafficking to late endosomes, VLPs consisting of the major EBOV structural proteins, GP, the matrix protein VP40, and nucleoprotein, were purified and DiI-labeled as described previously [9]. The HEK293 cells expressing enhanced green fluorescent protein fused to Rab7 (eGFP-Rab7), a late endosome marker was generated elsewhere [9]. HEK293 or HEK293 expressing eGFP-Rab7 cell lines were cultured in 35 mm glass-bottom dishes (MatTek Corporation) precoated with borate buffer containing 0.1 mg/ml poly-L-lysine (Sigma). The cells were inoculated with 1 μg/ml DiI-labeled VLPs treated with 20 μg/ml C1q, 10 μg/ml ZGP12/1.1, or combination of C1q and either ZGP12/1.1 or CTR IgG for 1 h at room temperature, followed by incubation for 30 min on ice. They were then incubated with phenol red-free RPMI containing 2% FCS and 4% bovine serum albumin (BSA) for 0 and 2 h at 37 ˚C to analyze DiI-labeled VLP attachment and trafficking to late endosomes, respectively. For Dextran uptake assay, HEK293 cells were incubated with phenol red-free RPMI containing 2% FCS, 4% BSA, and 0.5 mg/ml Dextran (Mw 10,000) labeled with Alexa Fluor 647 (Alexa647-labeled Dx10) (Molecular Probes) for 2 h at 37 ˚C. To count the number of DiI-labeled VLPs, the cells were fixed with 4% paraformaldehyde for 15 min at room temperature. Then the nuclei were stained with 1 μg/ml 4',6-diamidino-2-phenylindole, dihydrochloride (DAPI) (Molecular Probes) for 10 min at room temperature. Microscopic images were acquired using confocal laser-scanning microscope (Zeiss LSM780; Carl Zeiss) with a 63× oil objective lens and images were processed with the ZEN 2010 software (version 2.5; Carl Zeiss). For measurement of the number of DiI-labeled VLPs, images of 4–20 optical sections were acquired in 1 micron steps. The number of DiI-labeled VLPs was determined in approximately 100 individual cells using MetaMorph software (Molecular Devices) or Image J software (NIH) and the average number per cell was calculated for each condition. For colocalization analysis, the percentages of DiI-labeled VLPs that colocalized with eGFP-Rab7 (i.e., DiI/eGFP-double positive pixels/total DiI-positive pixels × 100) and DiI-labeled VLPs that colocalized with Alexa647-labeled Dx10 (i.e., DiI/Alexa647-double positive pixels/total DiI-positive pixels × 100) were measured in approximately 100 individual cells using the Coloc module in ZEN 2010 software (Carl Zeiss).

### Statistical analysis

All data were analyzed using Excel software. In all experiments, two-tailed paired Student’s *t*-test was used to evaluate statistical differences. *P* values of less than 0.05 were considered to be significant.

## Results and discussion

**Fig 1:**
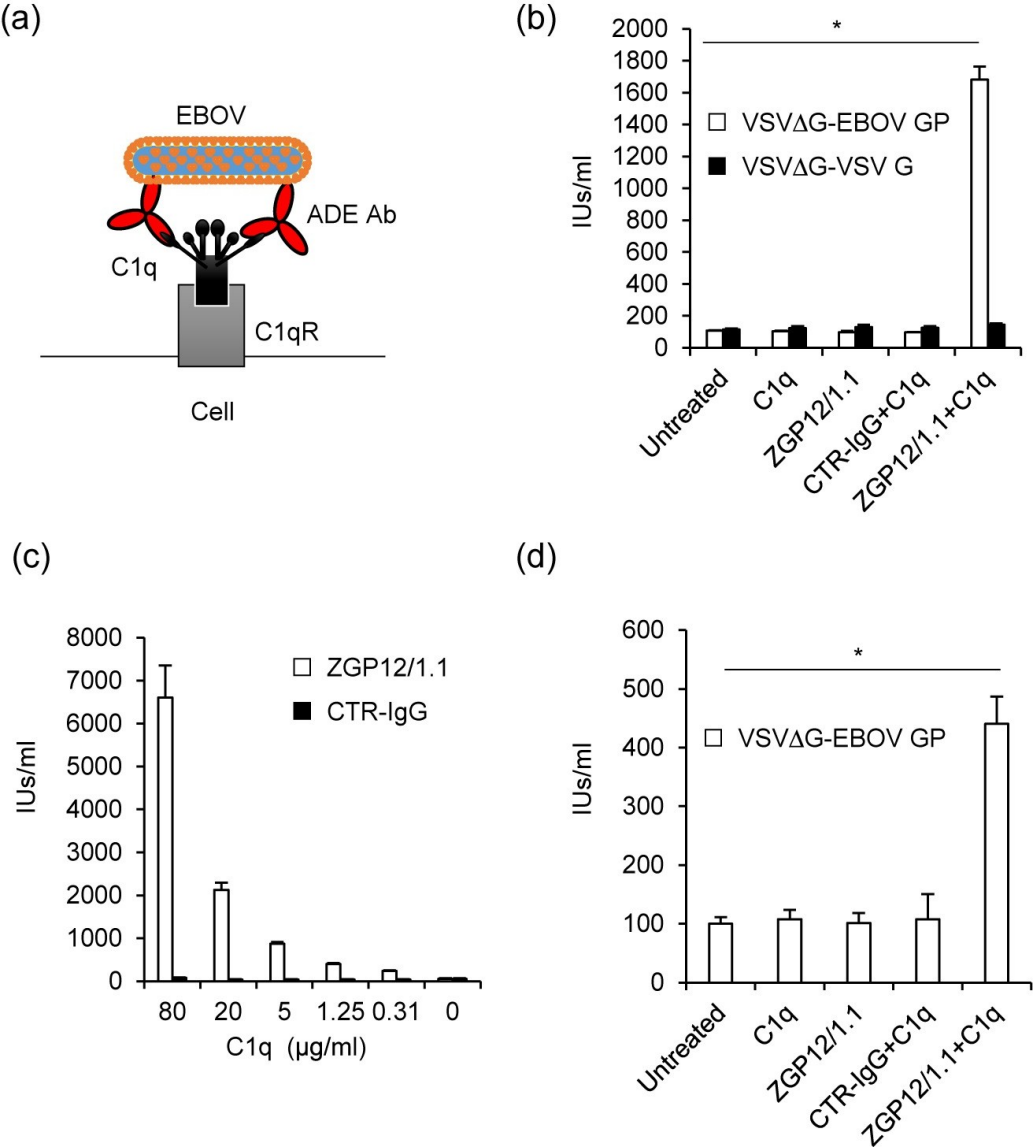
C1q-mediated ADE of EBOV GP-mediated infection. (a) Schematic representation of C1q-mediated ADE. (b) HEK293 cells were infected with VSVΔG-EBOV GP or VSVΔG-VSV G pretreated with C1q (20 μg/ml), ZGP12/1.1 (1 μg/ml), or combination of C1q (20 μg/ml) and either CTR-IgG (1 μg/ml) or ZGP12/1.1 (1 μg/ml). (c) VSVΔG-EBOV GP was incubated with ZGP12/1.1 (1 μg/ml) or CTR-IgG (1 μg/ml) together with the indicated concentrations of C1q and inoculated to HEK293 cells. (d) Vero E6 cells were infected with VSVΔG-EBOV GP pretreated with C1q (20 μg/ml), ZGP12/1.1 (1 μg/ml), or a combination of C1q (20 μg/ml) and either CTR-IgG (1 μg/ml) or ZGP12/1.1 (1 μg/ml). Virus titers were determined as IUs by counting GFP-positive cells. The mean and standard deviation of three independent experiments are shown. Statistical analysis was performed using Student’s *t*-test (*p < 0.05).

**Fig 2:**
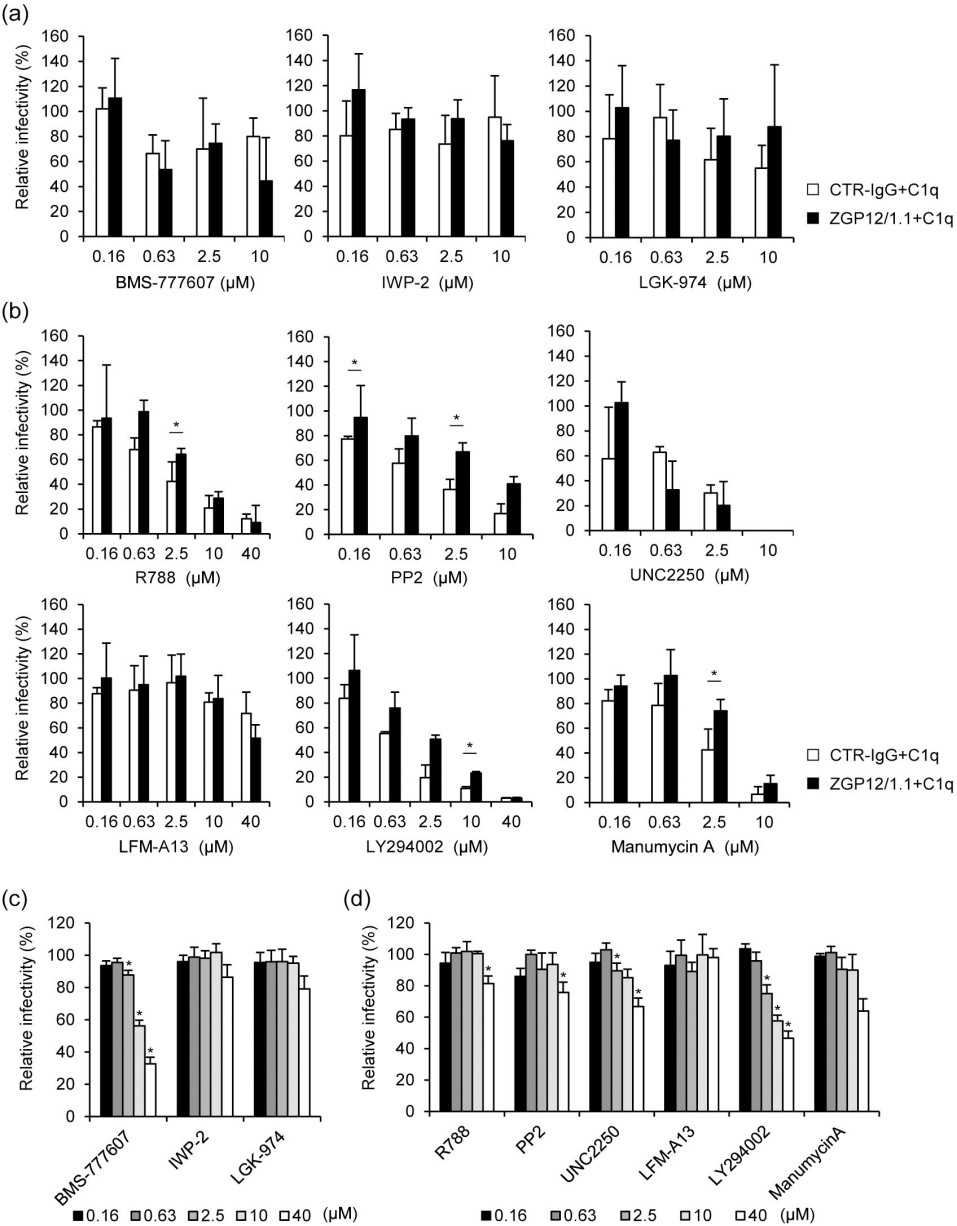
The effect of signaling pathway inhibitors on C1q-mediated ADE of VSVΔG-EBOV GP infection. (a, b) HEK293 cells were treated with the indicated concentrations of BMS-777607, IWP-2, LGK-974, R788, PP2, UNC2250, LFM-A13, LY294002, or manumycin A and infected with VSVΔG-EBOV GP preincubated with CTR-IgG (1 μg/ml) or ZGP12/1.1 (1 μg/ml) and C1q (20 μg/ml). (c, d) HEK293 cells were treated with the indicated concentrations of each inhibitor and infected with VSVΔG-VSV G. The relative percentage of infectivity was determined as described in Methods. The mean and standard deviation of three independent experiments are shown. Statistical analysis was performed using Student’s *t*-test (*p < 0.05). In panels c and d, statistical significances were evaluated for the comparison to control (DMSO- or ethanol-treated) cells.

**Fig 3:**
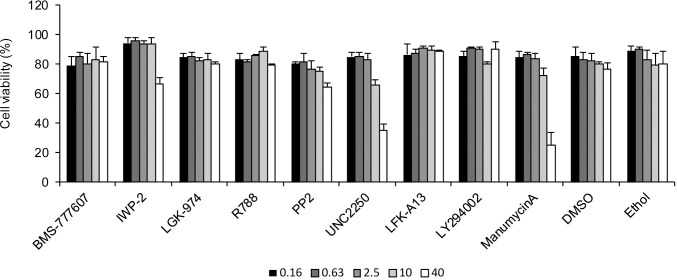
Cell viability in the presence of each inhibitor. HEK293 cells were treated with the indicated concentrations of each inhibitor. At 24 h after treatment, cell viability was determined using trypan blue staining.

Since our recent study has demonstrated that FcR-mediated ADE of EBOV infection depends on a cellular signaling cascade [9], we first hypothesized that C1q-mediated ADE would also use one or more signaling pathways that may be activated by the immune complex formed by EBOV, C1q, and C1q receptors (Fig 1A). In order to identify the intracellular signaling pathway involved in the C1q-mediated ADE of EBOV infection, we analyzed the effects of several inhibitors of signaling pathways in HEK293 cells. We first confirmed that the VSVΔG-EBOV GP titer, but not VSV-ΔG-VSV G, was significantly enhanced in the presence of combination of ZGP12/1.1 and C1q in HEK293 cells (Fig 1B). This effect was observed in a C1q dose-dependent manner (Fig 1C). The C1q-mediated ADE was also demonstrated in Vero E6 cells although the effect was lower than that seen in HEK293 cells (Fig 1D). We then tested chemical inhibitors of the Wnt/β-catenin signaling pathway (BMS-777607, IWP-2, and LGK-974) in the presence or absence of ZGP12/1.1 together with C1q in HEK293 cells since this signal is known to be involved in inducing C1q-mediated phagocytosis [17]. Unexpectedly, these signaling inhibitors blocked neither ADE nor non-ADE infection mediated by EBOV GP (Fig 2A). However, we showed that BMS-777607 reduced the infectivity of VSVΔG-VSV G in a dose-dependent manner, confirming that the inhibitor was active at the used concentrations (Fig 2C). Although the infectivity of VSVΔG-VSV G was not changed by IWP-2 and LGK-974, previous studies have shown that these inhibitors are effective at the experimental conditions used [18, 19]. Further testing of specific inhibitors targeting the spleen tyrosine kinase (R788), sarcoma family protein-tyrosine kinase (PP2), Mer tyrosine kinase (UNC2250), Bruton’s tyrosine kinase (LFM-A13), phosphatidylinositol 3-kinase (LY294002), and rat sarcoma (manumycin A) resulted in a similar reduction of both ADE and non-ADE infections with VSVΔG-EBOV GP in a dose-dependent manner (Fig 2B). The inhibitors showed no or little effect on the VSV G-mediated infection (Fig 2D) and cell viability (Fig 3). These results suggest that indeed some signaling pathways might be involved in the viral entry but that the tested inhibitors are not specific for the C1q-mediated ADE.

**Fig 4:**
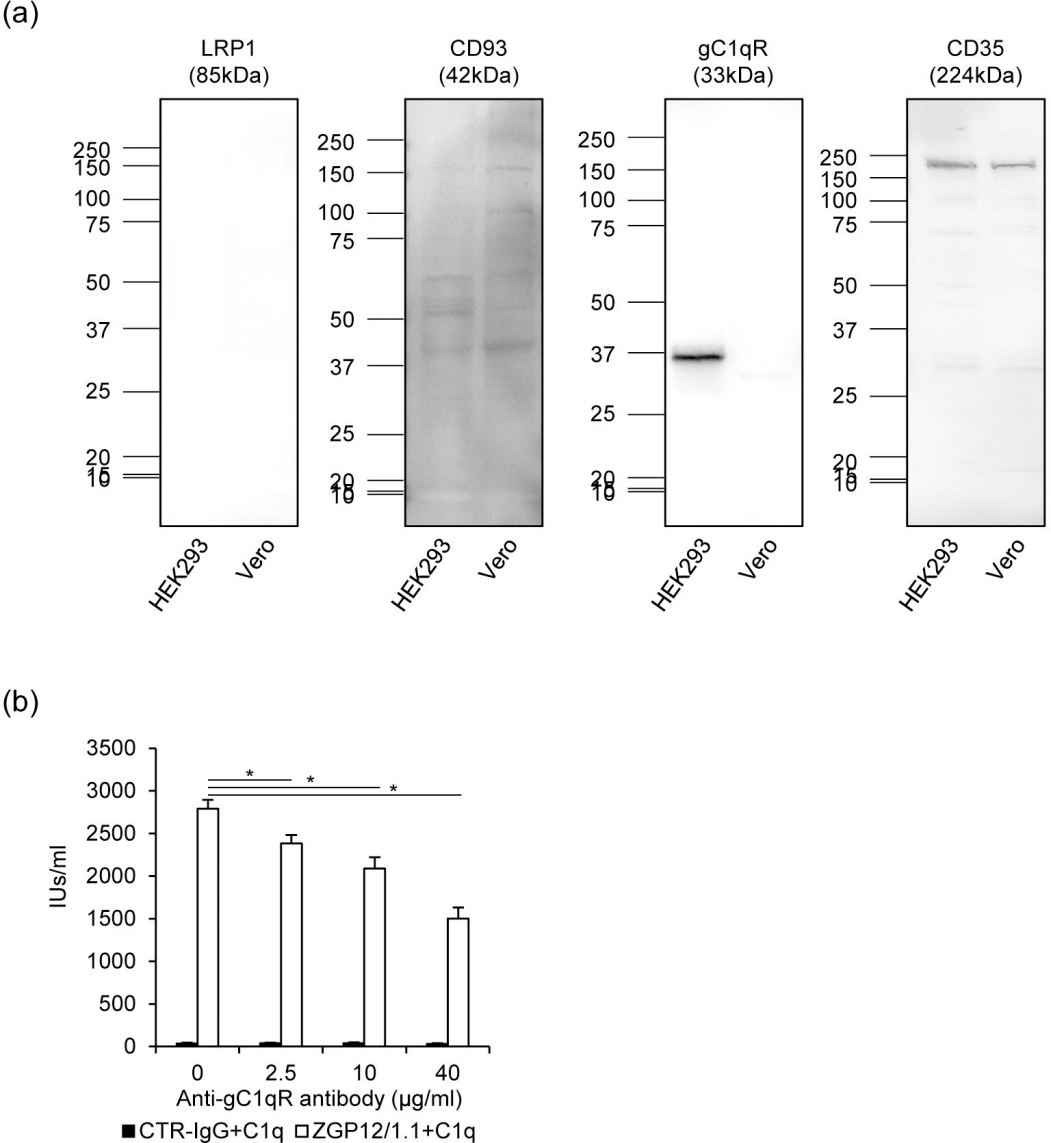
Expression of C1q receptors and contribution to C1q-mediated ADE. (a) The presence of C1q receptors LPR1, CD93, gC1qR and CD35 was confirmed by Western blot analysis of HEK293 and Vero E6 cell lysates. (b) HEK293 cells were treated with the indicated concentrations of anti-gC1qR antibody and infected with VSVΔG-EBOV GP preincubated with CTR-IgG (1 μg/ml) or ZGP12/1.1 (1 μg/ml) and C1q (20 μg/ml). The means and standard deviations of three independent experiments are shown. Statistical analysis was performed using Student’s *t*-test (*p < 0.05).

A number of complement receptors including CD35, CD93, gC1qR, and LRP-1 have been found and characterized [20]. First, we investigated which C1q receptors are expressed on HEK293 and Vero E6 cells using anti-C1q receptor antibodies (Fig 4A). We detected bands corresponding to CD35 and CD95 in the cell lysates, whereas LRP-1 was undetectable for both cell lines. Interestingly, gC1qR was only detected in HEK293 cells. It was previously shown that the anti-gC1qR antibody blocks C1q from binding to the receptor, however, the other antibodies have no blocking effect. We then found that the anti-gC1qR antibody significantly reduced C1q-mediated ADE in a dose-dependent manner most likely by blocking the interaction between C1q and gC1qR, although the antibody did not completely abolish the ADE effect (Fig 4B). These results indicate that the C1q-mediated ADE relies on C1q receptors including gC1qR.

Our previous study demonstrated that the FcR-mediated signaling pathway associated with the activation of sarcoma family protein tyrosine kinases was essential for EBOV ADE of infection [9]. In contrast to FcRs, which are expressed exclusively on immune cells such as monocytes and macrophages [21], C1q receptors have been identified in most mammalian cells [22], suggesting that one or more ubiquitous signaling pathways may contribute to C1q-mediated ADE of EBOV infection.

**Fig 5:**
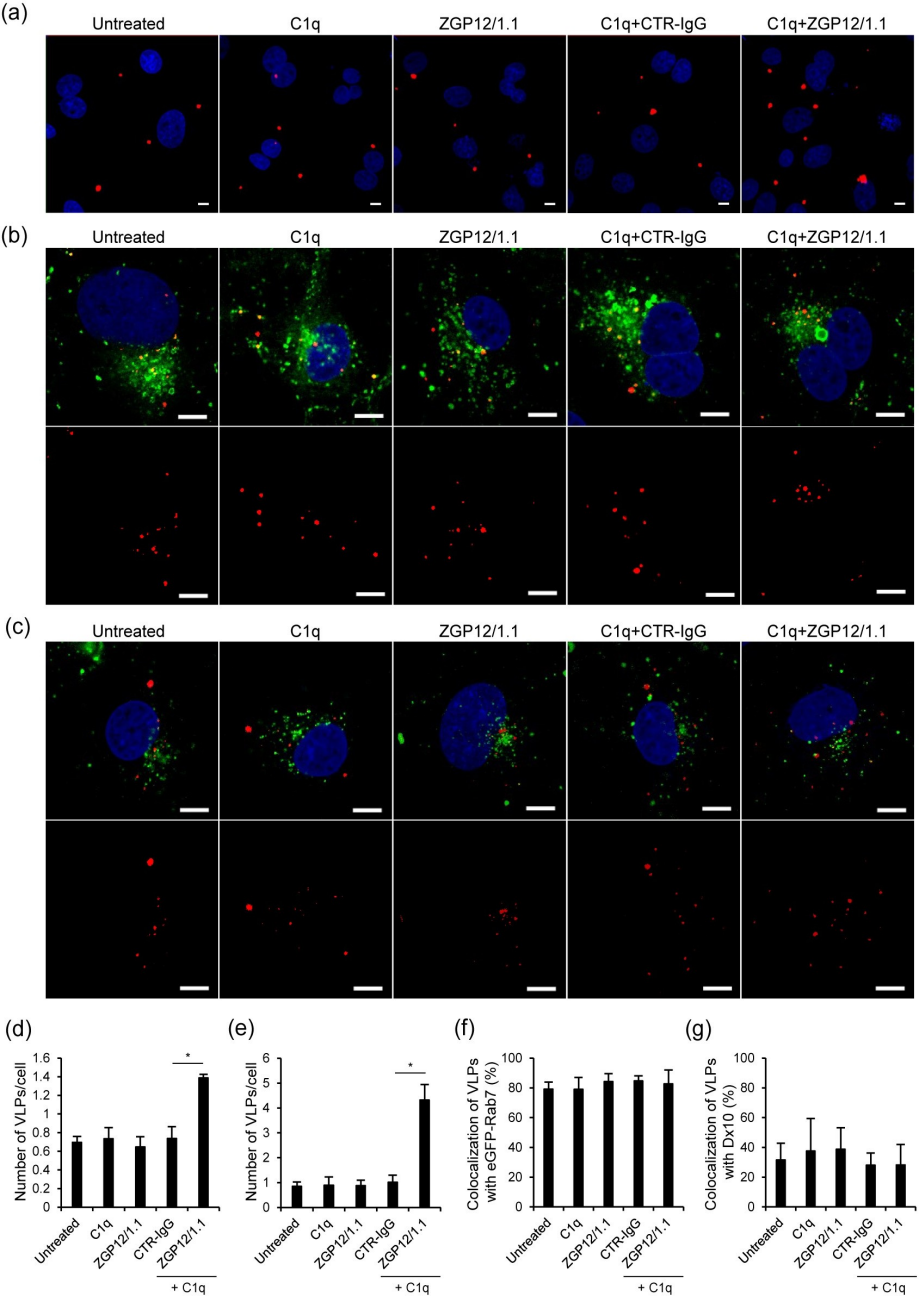
Enhanced VLP attachment to the cell surface in the presence of ZGP12/1.1 and C1q. (a, b, c) Fluorescent images of attachment and trafficking to late endosomes of VLPs. (d, e, f, g) Quantified fluorescent signals of VLPs, Rab7, and Dx10. HEK293 cells expressing eGFP-Rab7 (a, b) or HEK293 cells (c) were inoculated DiI-labeled VLPs mixed with PBS (Untreated), or treated with C1q (20 μg/ml) alone, ZGP12/1.1 (10 μg/ml) alone, CTR-IgG (10 μg/ml) and C1q, or ZGP12/1.1 and C1q. After adsorption, cells were incubated with Alexa647-labeled Dx10 (0.5 mg/ml) for 2 h at 37˚C (c). VLPs (red) on the cell surface at 0 h (a, d), VLPs (red) and eGFP-Rab7 (green) in the cytoplasm at 2 h (b, e, f), and VLPs (red) and Alexa647-labeled Dx10 (green) in the cytoplasm at 2 h (c, g) after adsorption were monitored by confocal laser scanning microscopy. Top panels show merged image and bottom panels show VLPs (b, c). Scale bars represent 10 μm (a, b, c). Nuclei of cells are visualized with DAPI (blue). The number of VLPs on the cell surface (d) and incorporated into the cells (e), the colocalization of VLPs (DiI) and eGFP-Rab7 signals (f), and the colocalization of VLP (DiI) and Dx10 (Alexa647) signals (g) were quantified and percentages of DiI-labeled VLPs that colocalized with eGFP-Rab7 (f) and Alexa647 labeled Dx10 (g) were determined as described in Methods. The mean and standard deviation of three independent experiments are shown. Statistical analysis was performed using Student’s *t*-test (*p < 0.05).

To investigate the mechanisms underlying C1q-mediated ADE of EBOV infection, we produced DiI-labeled VLPs and monitored the localization of VLPs in HEK293 cells. We first compared the number of VLPs attached to the cell surface after the treatment with C1q alone, the ZGP12/1.1 alone, CTR-IgG and C1q, or ZGP12/1.1 and C1q (Fig 5A and 5D). The number of VLPs attached to the cell surface was not significantly different between untreated, C1q-, and ZGP12/1.1-treated VLPs. In contrast, the attachment of VLPs was significantly enhanced by ZGP12/1.1, but not CTR-IgG, in the presence of C1q. Next, we compared the VLP uptake into cells using HEK293 cells expressing eGFP-Rab7, a late endosome marker. Although the number of VLPs incorporated into the cells was significantly enhanced by ZGP12/1.1 with C1q in consistent with the increased attachment of VLPs, the percentage of VLPs colocalizing with eGFP-Rab7 was not significantly different between any of the treatment groups, suggesting that cell-surface attached VLPs were taken up into endosomes under ADE and non-ADE conditions with similar efficiencies (Fig 5B, 5E and 5F).

Finally, we investigated whether C1q-mediated ADE of infection stimulates macropinocytosis, which has been shown to be the major pathway of EBOV entry into cells [23–25]. We measured the number of VLPs incorporated into intracellular vesicles along with Alexa647-Dx10, a specific probe for visualizing phagocytotic and macropinocytotic vesicles, as described previously [9, 23]. We found that approximately 30% of VLPs treated with ZGP12/1.1 and C1q overlapped with the Dx10 signals, however, this colocalization rate was not significantly different from the other treatment groups (Fig 5C and 5G).

It is known that EBOV entry is initiated by GP binding to cell surface attachment receptors such as T-cell immunoglobulin and mucin domain 1 (TIM-1) and C-type lectins [26–28], followed by internalization of virus particles into cells via macropinocytosis and subsequent membrane fusion mediated by the fusion receptor, Niemann-Pick C1 protein [23–25, 29, 30]. Taken together, our data suggest that cell surface C1q receptors act as an attachment factor like TIM-1 and C-type lectins and that C1q-mediated ADE of EBOV infection in HEK293 cells [4] depends on increased attachment of viral particles to the cell surface, most likely through direct binding of the virus-antibody-C1q complex to C1q receptors expressed on HEK293 cells. Although it is also possible that C1q sterically blocks the attachment of antibody-bound VLPs to TIM-1 and C-type lectins on cells lacking C1q receptors, it may not be a critical event in vivo since C1q receptors are present in most of cell types [20, 22].

The discovery of the C1q-mediated ADE mechanism provides a novel perspective for the general understanding of ADE of EBOV infection. Endothelial cells are not targeted by FcR-mediated ADE of infection, however, ADE of EBOV infection of endothelial cells has been suspected [4–6, 22]. With C1q being present in the plasma at a relatively high concentration, and C1q receptors being expressed in many cell types including endothelial cells, it is possible that the enhanced infection of endothelial cells by EBOV could be caused by C1q-mediated ADE of infection in the late stage of disease and may contribute to the exacerbation of the hemorrhagic manifestations. Furthermore, a recent study demonstrated that passive immunization with convalescent plasma is not associated with a significant improvement in survival of EBOV-infected patients, which might imply undesirable effects due to the presence of ADE antibodies [31]. Thus, the presence of ADE antibodies may be a potentially disadvantageous factor that should be considered when developing antibody-based therapeutics and prophylaxis against EBOV.
